# Exposures to Airborne Particulate Matter and Adverse Perinatal Outcomes: A Biologically Plausible Mechanistic Framework for Exploring Potential Effect Modification by Nutrition

**DOI:** 10.1289/ehp.9081

**Published:** 2006-08-17

**Authors:** Srimathi Kannan, Dawn P Misra, J. Timothy Dvonch, Ambika Krishnakumar

**Affiliations:** 1 Department of Environmental Health Sciences, Human Nutrition Program, School of Public Health, University of Michigan, Ann Arbor, Michigan, USA; 2 Department of Health Behavior and Health Education, School of Public Health, University of Michigan, Ann Arbor, Michigan, USA; 3 Department of Environmental Health Sciences, School of Public Health, University of Michigan, Ann Arbor, Michigan, USA; 4 Department of Child and Family Studies, Syracuse University, College of Human Studies and Health Professions, Syracuse, New York, USA

**Keywords:** air pollution, biomarkers, birth outcomes, cardiovascular disease, nutrition, particulate matter

## Abstract

**Objectives:**

The specific objectives are threefold: to describe the biologically plausible mechanistic pathways by which exposure to particulate matter (PM) may lead to the adverse perinatal outcomes of low birth weight (LBW), intrauterine growth retardation (IUGR), and preterm delivery (PTD); review the evidence showing that nutrition affects the biologic pathways; and explain the mechanisms by which nutrition may modify the impact of PM exposure on perinatal outcomes.

**Methods:**

We propose an interdisciplinary conceptual framework that brings together maternal and infant nutrition, air pollution exposure assessment, and cardiopulmonary and perinatal epidemiology. Five possible albeit not exclusive biologic mechanisms have been put forth in the emerging environmental sciences literature and provide corollaries for the proposed framework.

**Conclusions:**

Protecting the environmental health of mothers and infants remains a top global priority. The existing literature indicates that the effects of PM on LBW, PTD, and IUGR may manifest through the cardiovascular mechanisms of oxidative stress, inflammation, coagulation, endothelial function, and hemodynamic responses. PM exposure studies relating mechanistic pathways to perinatal outcomes should consider the likelihood that biologic responses and adverse birth outcomes may be derived from both PM and non-PM sources (e.g., nutrition). In the concluding section, we present strategies for empirically testing the proposed model and developing future research efforts.

Low birth weight (LBW) affects 20 million infants worldwide (United Nations International Children’s Fund 2006). LBW is comprised of two overlapping etiologies: preterm delivery (PTD) and intrauterine growth retardation (IUGR). LBW, IUGR, and PTD are all significantly associated with infant mortality and an array of infant morbidities that range from pulmonary to neurologic outcomes (Martin et al. 2002). These associations form the basis for the “fetal origins” or the “Barker hypothesis” which postulates that “fetal growth retardation consequent to malnutrition has long-term structural and physiologic impacts that predisposes an individual to chronic diseases in adulthood” (Barker and Fall 1998).

Perinatal outcomes are influenced by a multitude of factors including nutrition and health, genetics, physiologic stressors, and environmental toxicants such as ambient air pollution (Keen et al. 2003). In terms of the human health effects, the airborne particulate matter (PM) component has received the greatest attention (Šrám et al. 2005), and is therefore the focus for this review.

Current epidemiologic evidence suggests that maternal PM exposure is correlated with several adverse perinatal outcomes (Bobak 2000; Chen et al. 2002; Dejmek et al. 2000; Salam et al. 2005; Šrám et al. 2005; Wang et al. 1997; Wilhem and Ritz. 2005). Although these studies have become increasingly sophisticated in their measurement of PM exposures, the biologic roles of host factors that may function as effect modifiers of their relationship with birth outcomes have been less thoroughly examined. In particular, the lack of attention to nutrition factors should be considered. Nutrition can be both confounder and effect modifier of the associations between PM exposure and reproductive effects. Given the modifiable nature of both nutrition and PM exposures, future PM research and biomonitoring programs on young women would benefit greatly from the inclusion of selected nutrition factors. It is likely that women of childbearing age with nutritional risk factors (e.g., inadequate caloric intake, suboptimal protective antioxidant micronutrient status) are more likely to live in higher PM-exposed environments—confounded through their relation to socioeconomic status (SES) (Gwynn and Thurston 2001). Despite the considerable effects of nutrition among women of childbearing age, little is known about the nutrition interactions with SES and physical environment, such as PM exposure.

The specific objectives of this review are threefold: to describe the biologically plausible mechanistic pathways by which PM exposure may lead to adverse perinatal outcomes (LBW, IUGR, and PTD); review the evidence showing that nutrition affects the biologic pathways; and describe biologic markers that mediate the impact of nutrition and thereby explain the mechanisms by which nutrition may serve as effect modifiers of the association between PM exposure and perinatal outcomes.

## Responses to PM Exposures: Biologically Plausible Mechanisms

**Figure 1:**
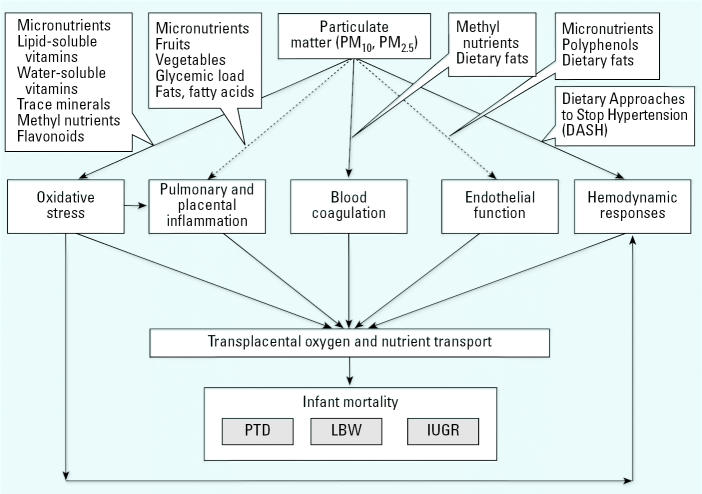
Proposed biologic framework for exploring possible effect modification of PM–birth outcomes by maternal nutrition.

The specific biologic mechanisms whereby PM influences perinatal outcomes remain to be fully elucidated. However, epidemiologic, clinical, and experimental evidence correlates current levels of PM with both respiratory and cardiovascular effects (Brook et al. 2004; Donaldson and MacNee 2001; Pope et al. 2004a, 2004b; Schwartz 2001), and provide corollaries around which we have developed biologically plausible hypotheses linking PM exposures and birth outcomes presented in Figure 1. Different particle size ranges including ultrafine particles (with aerodynamic diameter < 0.1 μm), fine particles (with aerodynamic diameter < 2.5 μm), and coarse particles (with aerodynamic diameter 2.5–10 μm) are of importance to this framework. Figure 1 illustrates both chronic and acute PM effects together. Five possible albeit not exclusive biologic mechanisms have been put forth in the literature to explain these effects. In the following text, we describe these mechanisms. Although an increasing number of studies support the notion that PM is associated with cardiovascular effects, these studies at present provide only a fragmentary and somewhat inconclusive picture of the complex biologic pathways involved.

### Oxidative stress

PM exposure may contribute to systemic oxidative stress (Donaldson and MacNee 2001) (Figure 1). Direct effects from oxidative activities of combustion-derived particles or by transition-metal constituents (e.g., iron, copper, chromium, and vanadium) (Adamson et al. 2000; Samet et al. 2000) may adversely affect the embryo in its earliest phase of growth (Mohorovic 2004). In addition, oxidative stressors resulting from PM exposure may arise from organic compounds and from activation of inflammatory cells capable of generating reactive oxygen species (ROS) and reactive nitrogen species (RNS) (Risom et al. 2005). F_2α_ (8-iso-PGF_2α_) isoprostane is one of the most promising biomarkers for assessing oxidative injury (Morrow et al. 1990) and has been studied the most extensively for PM exposures.

Oxidative stress–induced DNA damage appears to be a particularly important mechanism of action of urban particulate air pollution (Risom et al. 2005; Sorensen et al. 2003). As theorized by Hartwig et al. (2002), metals such as nickel in PM may inhibit DNA repair enzymes. We hypothesize that transplacental exposures to transition metals contained in PM could result in oxidative stress that may lead to DNA damage, disrupting DNA transcription which in turn may increase the number of placental DNA adducts. This hypothesis is partially supported by observations from the Czech Teplice study that found that maternal blood and placental DNA adducts are more common in areas with higher levels of air pollution (Topinka et al. 1997). One mechanism postulated to mediate the effects is that PM absorbs and transports polycyclic aromatic hydrocarbons (PAHs), exposure to which may lead to increased DNA adducts (Perera et al. 1998, 1999), thus resulting in LBW (Perera et al. 1998, 1999) and IUGR (Dejmek et al. 1999, 2000). Researchers suggest that DNA damage measured by oxidized DNA bases purines and pyrimidines and protein and lipid peroxidation indicated by plasma malondialdehyde may be more sensitive than bulky DNA adducts as markers of exposure to PM (Risom et al. 2005). PAHs in PM can induce biotransformation by cytochrome P450, expoxide hydrolase, and dihydrodiol dehydrogenase (Burczynski et al. 1999) in addition to the direct action of coal combustion toxics on antioxidants/enzymes (e.g., superoxide dismutase, catalase) that may adversely affect the embryo in its earliest phase of growth (Mohorovic 2004). Alternatively, PM may also bind receptors for placental growth factors, resulting in decreased fetal–placental exchange of oxygen and nutrients (Dejmek et al. 2000). Nutrient and oxygen supply during gestation are key factors regulating fetal growth (Harding and Johnston 1995).

### Pulmonary and placental inflammation

PM exposure is associated with systemic inflammation (Brook et al. 2003; Panagiotakos et al. 2004; Peters et al. 2001; Pope et al. 2004b; Seaton et al. 1999) (Figure 1). We hypothesize that inhalation of particles during pregnancy can induce acute placental (Bobak 2000) and pulmonary inflammation. In contrast to the PM composition–induced effects on oxidative stress that have been extensively studied, specific components in particles that elicit inflammation are less thoroughly investigated, although recent research points to the contribution of compositional trace elements (Saldiva et al. 2002) and bioavailable transition metals to cardiopulmonary injury in healthy and compromised animal models (Costa and Dreher 1997). Based on cell culture methodologies, the up-regulation of pro-inflammatory mediators in response to transition metals chromium, aluminum, silicon, titanium, iron, and copper within PM were found to contribute to pulmonary inflammation (Risom et al. 2005).

The most widely studied biomarkers of inflammation are high-sensitive C-reactive protein, oxidized low-density lipoproteins, proinflammatory cytokines interleukin (IL)-1, IL-6, and tumor necrosis factor-α, serum amyloid A (Pearson et al. 2003), the acute phase marker fibrinogen, neutrophil count and blood platelet count, red blood cells and white blood cells (Seaton et al. 1999), and albumin (Liao et al. 2005). With cell culture methods, PM exposure–induced trace elemental markers of inflammatory response denoted by the release of cytokines and chemokines were recently identified by Becker et al. (2005), who showed that PM constituent iron and silicon correlated with the release of IL-6, whereas chromium correlated with IL-8.

Inflammation could be associated with inadequate placental perfusion (Knottnerus et al. 1990), which can mediate placental inflammatory responses and its biologic sequelae, resulting in impaired transplacental nutrient exchange (Bobak 2000) (Figure 1). We hypothesize that inadequate placental perfusion may cause growth restriction *in utero* due to interference with some process or processes such as affecting nutrition of the fetus, reduced oxygenation of maternal blood, or both. For example, a rapid decline in the placental delivery of essential fatty acids arachidonic acid and docosahexanoic acid is expected (Crawford 2000).

Independent of the cascade of events characterized above, the biologic mechanisms that trigger adverse perinatal outcomes may include maternal infections, especially during the last trimester of pregnancy, and may initiate premature contractions and/or rupture of membranes (Wilhelm and Ritz 2005). Although air pollution does not directly cause maternal infections, exposure to specific pollutants may enhance allergic inflammation (Nel et al. 1998) and increase the maternal risk for adverse birth outcomes.

### Coagulation

Systemic alterations in rheologic factors, including blood coagulability and whole blood viscosity as a result of exposure to PM, represent other potential mechanisms of PM toxicity (Pekkanen et al. 2000; Peters et al. 1997; Prescott et al. 2000; Seaton et al. 1999). In response to PM exposures, increase in any of the proteins of the clotting cascade present a possibility for coagulation (Donaldson and MacNee 2001; Pekkanen et al. 2000). Based on a cross-sectional study conducted in London, Pekkanen et al. (2000) found ambivalent results for the association between PM_10_ (PM < 10 μm in aerodynamic diameter) and plasma fibrinogen—this association was significant only for the warm season. Other measurable biomarkers include factors VII–IX, fibrin d-dimer, and von Willebrand factor (Jansson et al. 1991). PM exposures may also lead to changes in hemoglobin, platelets, and white blood cells (Riediker et al. 2004), which may potentially contribute to the association between PM and adverse fetal growth.

### Endothelial function

Exposure to PM may influence endothelial functions and could be considered as an intervening pathway in subsequent impact on fetal growth (Figure 1). Although this pathway has been less extensively studied, the impact of PM on vascular function has been the subject of recent investigations (Brook et al. 2003). Inhalation of environmental tobacco smoke (ETS) [similar in characteristics to PM_2.5_ (PM < 2.5 μm in aerodynamic diameter)] causes rapid vasoconstriction (Ambrose and Barua 2004), increases plasma endothelin levels (Goerre et al. 1995), and triggers endothelial dysfunction (Otsuka et al. 2001). Although the specific chemical components of ETS responsible for the observed effect of vasoconstriction have not been adequately characterized, it is likely that the PM in ETS is primarily responsible, as summarized by Brook et al. (2004).

A recent animal-based study (Dvonch et al. 2004) found that PM_2.5_ exposure increased plasma concentrations of asymmetric dimethyl arginine that is associated with impaired vascular function and increased risk of cardiovascular events (Valkonen et al. 2001). Circulating concentrations of soluble adhesion molecules E-selectin, intracellular adhesion molecule (sICAM-1), and vascular cellular adhesion molecule (VCAM-1) are overexpressed when the endothelium encounters inflammatory stimuli (Hwang et al. 1997). The inhalation of high urban levels of concentrated ambient particles and ozone for 2 hr caused conduit arterial vasoconstriction in healthy adults (Brook et al. 2002). As summarized by Brook et al. (2004), it is possible that acute systemic inflammation and oxidative stress following PM exposure (Sorensen et al. 2003) are responsible for triggering endothelial dysfunction leading to vasoconstriction (Bonetti et al. 2003). Endothelial dysfunction can also be secondary to other cardiovascular disease (CVD) risk factors (e.g., metabolic syndrome) (Roberts et al. 2003). These pathophysiologic reactions in response to PM exposures may result in impaired fetal growth.

### Hemodynamic responses

Biologic measures that assess hemodynamic changes in response to PM exposure have typically included systolic blood pressure (SBP) and diastolic blood pressure (DBP). Panel studies conducted of adults with preexisting CVD found an increase in SBP associated with elevated particulate exposures (Ibald-Mulli et al. 2001; Linn et al. 1999; Zanobetti et al. 2004) (Figure 1). In contrast, based on population exposures, an increase of a 5-day average of ultrafine particles was associated with a small decrease in SBP and DBP (Ibald-Mulli et al. 2004). Specific biologic mechanisms for the observed PM-associated effects on blood pressure (BP) have been suggested to include an increase in sympathetic tone and/or the modulation of basal systemic vascular tone (Ibald-Mulli et al. 2001). Another potential mechanism whereby pollutant components can increase BP is superoxide-mediated inhibition of the actions of nitrous oxide in inducing vasodilatation (Delfino et al. 2005).

If PM exposure is also associated with BP elevations in pregnant women, this could increase the risk of adverse perinatal outcomes as a consequence of preexisting hypertension or pregnancy-induced hypertension. Elevation of BP to levels that is defined as pregnancy-induced hypertension has been associated with IUGR (Misra 1996) and PTD (Misra 1996). Severely impaired fetal growth is preceded by maternal hemodynamic maladaptation (Duvekot et al. 1995). These changes may force the fetus to adapt, down-regulate growth, and prioritize the development of essential tissues (Fall et al. 2003). Hypertension can also be secondary to oxidative stress and vascular inflammation (Virdis and Schiffrin 2003) or other risk factors, for low maternal body weight, for example (Ehrenberg et al. 2003), thus enhancing the susceptibility to adverse birth outcomes.

## Exploring Effect Modification by Nutrition

Although the specific underlying mechanisms that contribute to normal or adverse birth outcomes are not yet fully understood, an adequate periconceptional nutrition status is considered a key determinant (Henriksen and Clausen 2002; Hobel and Culhane 2003). Given that both dietary composition and CVD risk are strongly socially patterned, this suggests one way to approach the possible interaction between air pollution and SES (in affecting birth outcomes). As is described in more detail below and illustrated in Figure 1, dietary composition has been demonstrated to relate to those same biologic mechanisms hypothesized to explain the possible effects of PM exposure on birth outcomes.

The nutrition aspects of the framework shown in Figure 1 are not intended to include every possible parameter worthy of consideration. Explorations about what to add to various layers of the framework could be one of its most useful applications in future work on this topic. Although no previous studies of the perinatal effects of PM exposure have examined effect modification by nutrition, theoretical and empirical evidence is growing. Researchers studying air pollution and birth outcomes have suggested that nutrition status may play a role in protecting the fetus or magnifying the effects (Dejmek et al. 1999; Ritz and Yu 1999). Other investigators have cited the potential importance of nutrition as a buffering or synergistic factor with regard to PM-induced cardiovascular responses (Hennig et al. 2005; Ostro et al. 2006; Schwartz 2001). Using data from the Third National Health and Nutrition Examination Survey (NHANES III), Schwartz (2001) considered the role of nutrition in the association between PM exposures and incident ischemic events. Considering a limited set of dietary factors (saturated fat, fiber, alcohol, caffeine, fish and shellfish), Schwartz (2001) reported that the selected factors did not modify the association. Furthermore, the biomarkers were limited to fibrinogen, platelet and white blood cell count, SBP, total cholesterol, and high-density lipoprotein cholesterol. On the other hand, we propose that researchers should explore the potential effect-modifying roles using a more comprehensive list of dietary variables and biomarkers.

## Consideration of a Hypothesis of Nutritional Susceptibility

The Institute of Medicine (1999) describes combinations of environmental exposures and greater susceptibility as a form of “double jeopardy.” Maternal nutrition stressors such as micronutrient deprivation are likely to occur around the world in subpopulations that experience disparate air pollution profiles. Considerable research evidence supports the important role played by nutrition, particularly micronutrients, in determining positive pregnancy outcomes (Black 2001). In addition, gestational energy stress, a phenomenon characterized by lower plasma volume expansion (Mardones-Santander et al. 1999), protein-energy malnutrition, and pregnancy complications, may also co-occur. As depicted in Figure 1, we propose that maternal nutrition could be exacerbating or buffering in the association between PM and birth outcomes for a subgroup of women of childbearing age. In the following section, we contextualize these biologic pathways for nutrition: first based on intakes of nutrients, next based on the consumption of foods or of groups of foods, and finally based on indices and dietary patterns that combine both approaches (Kant 1996).

### Nutrients potentially contributing to biologic pathways

In the past two decades, understanding of cardioprotective nutrients and foods has grown substantially owing to studies of the molecular mechanisms and the metabolic effects. Investigators typically estimate nutrient intakes using food frequency questionnaires (Block et al. 2001; Kristal et al. 2000; Willett 1989), food records, and/or 24-hr dietary recalls. Nutrient values may be derived using existing databases (U.S. Department of Agriculture 1992) supplemented with information from manufacturers and biochemical analyses.

#### Oxidative stress

Ingestion of particular micronutrients causes a shift in oxidative status. The micronutrients most relevant to the pathways shown in Figure 1 include the fat-soluble carotenoids and vitamin E, water-soluble vitamin C, (Mayne 2003) and methyl nutrients including the B-vitamins pyridoxine (B_6)_, cyanocobalamin (B_12_), and folate. Carotenoids may protect against oxidant damage (Porrini et al. 2002). Dietary micronutrient trace minerals zinc and manganese may display indirect antioxidant activity as constituents of enzymes including superoxide dismutase. Micronutrients may extend the gestational period to full term or counteract the damage caused to lipids and DNA triggered by PM exposures (Smolkova et al. 2004). Methyl nutrients are involved in DNA methylation (Ames 1999), and the resulting methyl nutrient status may modify PM-induced alterations in oxidative stress through its impact on DNA stability, repair, and the different gene expression processes. Suboptimal methyl nutrient status may also increase the risk for PTD associated with preeclampsia (Powers et al. 1998) and LBW (Vollsett et al. 2000).

#### Inflammation

Dietary macronutrient intakes may produce inflammatory responses. Unlike micronutrients, some macronutrients may show opposite effects. Reducing *trans*- and saturated fatty acids and increasing omega-3 fatty acids are also associated with a reduced inflammatory status. Food sources rich in n-6 polyunsaturated fatty acids are shown to enhance IL-1 production; n-3 fatty acids on the other hand have been demonstrated to have the opposite effect (Lopez-Garcia et al. 2004).

#### Coagulation

A deficiency in any one of the methyl nutrients could result in elevated homocysteine (McCully 1993). Homocysteine thiolactone can subsequently influence vascular coagulation (McCully 1993). In addition, high total dietary fat may lead to fibrin deposits and thrombus formation through activation of coagulation (Miller 2005).

#### Endothelial function

Micronutrient antioxidants representing β-carotene subfractions derived from vegetables and fruits are inversely related to E-selectin (Rowley et al. 2003). Polyphenols have been found to inhibit expression of endothelial adhesion by regulating gene transcription (Carluccio et al. 2003). Micronutrient intakes such as arginine and folic acid have been shown to improve endothelial function (Cuevas and Germain 2004). Unlike the possible cardioprotective effects of micronutrients and polyphenols, macronutrients may be beneficial or detrimental. Based on the Nurses Health Study, Lopez-Garcia et al. (2004) reported a positive relationship between *trans*-fats and endothelial dysfunction, whereas n-3 fatty acids were inversely associated with sICAM-1, sVCAM-1, and E-selectin.

#### Hemodynamic responses

The favorable effects of fruits and vegetables, low-fat dairy products, and reduced sodium suggested by Dietary Approaches to Stop Hypertension (DASH) (Appel et al. 1999) indicate the possible role for micronutrients in reducing the risk for prepregnancy hypertension. Several mechanisms of polyphenols have been researched, including their antioxidant functions.

### Contributions of foods/food groups to biologic pathways

There is a growing list of foods and food groups consumption of which is associated with the various biologic pathways depicted in the present framework (Figure 1). Fruits and vegetables contain a myriad of different components of varying antioxidant capacity, thus offering a range of possibilities for altering PM-induced oxidative effects (O’Byrne et al. 2002). Based on the NHANES III findings, grain consumption is inversely associated with an elevated CRP concentration (Ford et al. 2005). Similarly, fresh fruit, olive oil, mushrooms, cruciferous vegetables, and nuts are associated with a favorable homocysteine profile (Weikert et al. 2005). Adding vegetables may reverse the increases in ICAM-1 and VCAM-1, whereas high intakes of refined grains, and processed meat and low consumption of cruciferous and yellow vegetables may exacerbate the inflammatory processes (Giugliano et al. 2001).

### Dietary patterns as contributors to the biologic pathways

Dietary pattern analysis serves as a complementary approach to the nutrient-focused and food-group analysis described above. Dietary patterns are food intake patterns over a referent period and consider the overall dietary matrix (Fung et al. 2001; Hu 2002; Kerver et al. 2003; Tseng and DeVillis 2003). However, most of these studies did not focus on the dietary patterns among women of childbearing age.

Dietary patterns cannot be measured directly, and one must rely on statistical methods that employ dimension-reduction techniques such as factor analysis and cluster analysis (Fung et al. 2001). The advantage of novel statistical approaches such as the reduced rank regression (Hoffman et al. 2004) is that the derived pattern incorporates the biologic pathways presented in the current framework and thus is hypothesis driven.

### Gene–nutrient interactions and impact on biologic pathways

Nutrigenomic researchers have provided evidence for interactions among dietary factors, genetic variants, and biochemical markers of CVD (Ordovas 2004). Genetic background can interact with habitual total dietary fat and fatty acid composition, thereby affecting predisposition to the woman’s responsiveness to PM exposures. Similarly, genetic susceptibility related to functional polymorphisms in genes coding for antioxidant and DNA repair enzymes may be expected to modify the levels of oxidative DNA damage caused by exposure to PM. In addition, there is significant evidence that genes are involved in determining enzymes, receptors, cofactors, and structural components involved in regulation of BP and inflammatory and coagulation factors (Ordovas 2004).

## Measurement Indices for Nutrients, Foods, and Food Groups and Dietary Patterns

Individual dietary constituents may have small biologic effects that emerge only when the components are integrated into a simple unidimensional score. Appendix 1 lists candidate tools, and we have classified them in three categories as a function of their determination mode, based on a now classical review (Kant 1996): *a*) indices based on intakes of nutrients (or at least of certain nutrients); *b*) indices based on the consumption of foods or of groups of foods; and *c*) indices that combine both approaches resulting in dietary patterns. In the following section, we present examples of these measurement indices that add quantitative elements to qualitative aspects, and some are based on thresholds or recommendations. In a few cases, the indices were studied to link to the biologic parameters in the present framework.

### Nutrient indices

Oxidative stress has been described as a disturbance in the balance between free radical production and antioxidant capacity (Ames 1999). Reflecting this definition, the dietary antioxidant index summarizes the combined intakes of carotenoids, flavonoids, tocopherols, tocotrienols, selenium, and vitamin C (Wright et al. 2004). The integrated oxidative balance score reflects antioxidant (e.g., vitamin C) and pro-oxidant (e.g., iron) intakes (Van Hoydonck et al. 2002). The antioxidant scores for commonly consumed fruits, fruit juices, and vegetables are published as oxygen radical absorbance capacity (ORAC) or ferric-reducing antioxidant power (Cao et al. 1993; Cao et al. 1996). More than 80% of the antioxidant capacity in fruits and veggies may also be attributed to flavonoids (Peterson and Dwyer 1998) that have the ability to chelate metal ions (Belguendouz et al. 1997) and have particular relevance here.

### Foods and food group indices

Dietary variety determined by Recommended Foods score (simple count of consumed food items) and diversity measured as Dietary Diversity Score (count of represented food groups) (Kant 1996) are both good candidates for measuring overall dietary quality. The Healthy Eating Index based on the Dietary Guidelines for Americans is an additional measure of quality (Kennedy et al. 1995). The Mediterranean pattern now recommended for the secondary prevention of coronary artery disease quantifies adherence to the traditional Mediterranean diet using a 9-point scale (Trichopoulou et al. 2003). Minor variants to these indices, the alternate Healthy Eating Index (Fung et al. 2005) and the alternate Mediterranean dietary pattern (Fung et al. 2005) were found to be associated with markers of inflammation.

### Dietary pattern indices

The possibility that dietary patterns may exert an effect on biologic measures was first suggested through the findings of the DASH clinical trial (Appel et al. 1999) (Appendix 1). As shown in Appendix 1, other population studies conducted in the United States indicate two major dietary patterns: “prudent” and “Western” (Tseng and De Villis 2000). The prudent pattern was found to be inversely associated with homocysteine and positively associated with folate (Fung et al. 2001) while also showing a beneficial effect on the endothelium. The Western pattern, on the other hand, was positively correlated with homocysteine, high-sensitive C-reactive protein, and impaired endothelial function and negatively associated with folate (Martinez-Gonzalez and Sanchez-Villegas 2004). Similarly, low-glycemic load-based patterns in women of child-bearing age were associated with improved fibrinolysis (Jarvi et al. 1999). Glycemic load may be determined using the updated table that provides glycemic index scores for 1,300 international food entries (Foster-Powell et al. 2002) (Appendix 1).

## Recommendations Related to the Proposed Framework

In Appendix 2, we recommend strategies for developing future research efforts in three overarching areas. Certainly many factors could function as mediators of the association between PM and birth outcomes. However, few studies are sufficiently comprehensive to understand the multifactorial etiologies and pathways. In particular, the confounding nature of SES and air pollution should be explored in future work. Future studies that include biomarkers of exposure/effect and are informed by biologic pathways will help tease out those aspects of SES that explain differences in PM birth effects among population subgroups.

The current framework may be advanced by biomonitoring women with unique circumstances (e.g., genetic polymorphisms). Further research will help identify susceptible population subgroups, such as for the potential for genetic variation in metabolic pathways (e.g., detoxifying enzymes such as cytochrome P450) that could underlie differences in susceptibility to toxicities related to PM exposures (Perera et al. 1999; Šrám 1998). Altered expressions of DNA repair and other defense genes have yet to be studied for up-regulation of the involved enzymes that may alleviate effects of repeated PM exposures (Risom et al. 2005).

The available data are consistent with the occurrence of PM-related systemic oxidative, inflammatory, and hemodynamic responses, but evidence on endothelial dysfunction and procoagulatory states is limited. In addition to these pathways, other alternate mechanisms (e.g., disruption in iron homeostasis) (Ghio and Cohen 2005) should be studied. Although mechanisms underlying the adverse effects of PM on the cardiopulmonary systems remain a primary focus of research, additional hypotheses suggest the involvement of neurogenic processes (Campbell et al. 2005; Pope et al. 2004b). Finally, researchers should also consider the synergistic interactions among the various biologic mechanistic pathways.

## Conclusion

Several ongoing U.S. population-based research projects funded through the National Institute of Environmental Health Sciences (e.g., the Health Disparities Initiative) provide unique opportunities to apply and evaluate the current framework. The resulting findings would be relevant for PM regulation and primary prevention of CVD and other diseases influenced by the pathways proposed in the current framework and reducing the risks for adverse birth effects. If exposure interactions are found for PM with nutrition, they may also offer geographically relevant nutrition–environment interactions-based intervention opportunities through various federal food and nutrition assistance venues including the Special Supplemental Nutrition Program for Women, Infants, and Children.

## Appendix 1. Measurement Indices Assessing Nutrients, Foods, Food Groups, and Dietary Patterns

### Dietary Intakes of Nutrients

- ***Dietary Antioxidant Index*** (Wright et al. 2004): carotenoids, flavonoids, tocopherols, tocotrienols, selenium, and vitamin C
- ***Oxidative Balance Score*** (Van Hoydonck et al. 2003): vitamin C, β-carotene, and iron
- ***Oxygen Radical Absorbance Capacity (ORAC)*** (Cao et al. 1993, 1996): ORAC_ROO_ (peroxyl radical), ORAC**·**_OH_ (hydroxyl radical), and ORAC_Cu_ (copper)

### Dietary Intakes of Foods and Food Groups

- ***Dietary Diversity Score*** (Kant 1996): foods from dairy, meat, grain, fruit, and vegetable groups
- ***Recommended Foods Score*** (Kant 1996): weekly consumption of fruits, vegetables, lean poultry and alternates, low-fat dairy, and whole grains

### Combined Dietary Intakes of Nutrients, Foods, and Food Groups

- ***Dietary Approaches to Stop Hypertension*** (Appel et al. 1999): 4–5 servings fruits; 4–5 servings vegetables; 2–3 servings low-fat dairy products; 7–8 servings of grain products; 2 or less servings of meats, poultry, fish/day; 4–5 servings of nuts, seeds, legumes/week
- ***Alternate Healthy Eating Index*** (Fung et al. 2005): protein source, *trans* fat, PFA:SFA, cereal fiber, moderate alcohol, and long-term multivitamin use
- ***Alternate Mediterranean Diet*** (Fung et al. 2005): excludes potato products from vegetable group, separates fruit and nuts into two groups, eliminates dairy group, includes “whole grain” products only, only red and processed meats for meat group
- ***Prudent Pattern*** (Tseng and De Villis 2000): fruits, vegetable, fish, whole grains, and legumes
- ***Western Pattern*** (Tseng and De Villis 2000): red and processed meat, high-fat dairy products, sugar-containing beverages, sweets, and desserts
- ***Glycemic Load*** (Foster-Powell et al. 2002): glycemic quality and quantity

## Appendix 2. Recommendations for Advancing the Current Framework

### Sampling, Measurement, and Characterization of PM Exposures

- Consider the roles of co-pollutants (e.g., ozone, carbon monoxide, nitrogen dioxide) with PM and use multiple-influence chemical characterization models.
- Incorporate trace elements that are characteristic to their specific source type and emissions through specific source “fingerprints.”
- Integrate personal PM exposures with fixed-site and community-level assessment.
- Consider the geographical and seasonal toxicity profiles for PM and constituents.
- Collect continuous ambient PM exposure over and beyond “daily” PM data.
- Explore the intracellular pathways by which PM and constituent transition metals may modulate the gene expression of biologic responses.

### Assessing Nutritional Status, Biologic Pathways, and Biomarkers of Response

- Explore the possible dietary influences by incorporating *a priori* approach, which builds on previous knowledge concerning the cardiac and pulmonary effects, and birth outcomes.
- Assess specific food features, depending on the contexts relevant to PM monitoring area, and construct dietary indices accordingly.
- Enhance the reliability and validity of self-reported nutrition measures by incorporating relevant biologic measures.

### Clarifying the Temporal and Spatial Vulnerabilities and Unique Circumstances

- Expand the proposed framework using the definition of maternal health that fosters linkages with a woman’s health during her reproductive years.
- Consider all gestational time windows of PM and *in utero* nutrition exposures.
- Explore susceptibility resulting possibly from compromised maternal health, in addition to effects exerted directly across the placenta.
- Clarify the roles of multiple determinants (SES and other stressors) in causing adverse birth outcomes.
- Biomonitor women for gene polymorphisms (gene–gene, gene–nutrient, gene–nutrient–environment) by which PM and constituent transition metals may modulate the gene expression of biological responses.

## References

[b1-ehp0114-001636] Adamson IY, Prieditis H, Hedgecock C, Vincent R (2000). Zinc is the toxic factor in the lung response to an atmospheric particulate sample. Toxicol Appl Pharmacol.

[b2-ehp0114-001636] Ambrose JA, Barua RS (2004). The pathophysiology of cigarette smoking and cardiovascular disease: an update. J Am Coll Cardiol.

[b3-ehp0114-001636] Ames BN (1999). Micronutrient deficiencies. A major cause of DNA damage. Ann NY Acad Sci.

[b4-ehp0114-001636] Appel LJ, Vollmer WM, Obarzanek E, Aicher KM, Conlin PR, Kennedy BM (1999). Recruitment and baseline characteristics of participants in the Dietary Approaches to Stop Hypertension trial. DASH Collaborative Research Group. J Am Diet Asso.

[b5-ehp0114-001636] Barker DJP Fall CHD 1998. The Immediate and Long-Term Consequences of Low Birthweight. Technical Consultation on Low Birthweight. New York:UNICEF.

[b6-ehp0114-001636] Becker S, Dailey LA, Soukup JM, Grambow SC, Devlin RB, Huang YCT (2005). Seasonal variations in air pollution particle-induced inflammatory mediator release and oxidative stress. Environ Health Perspect.

[b7-ehp0114-001636] Black RE (2001). Micronutrients in pregnancy. Br J Nutr.

[b8-ehp0114-001636] Block G, Norkus E, Hudes M, Mandel S, Helzlsouer K (2001). Which plasma antioxidants are most related to fruit and vegetable consumption?. Am J Epidemiol.

[b9-ehp0114-001636] Bobak M (2000). Outdoor air pollution, low birth weight, and prematurity. Environ Health Perspect.

[b10-ehp0114-001636] Bonetti PO, Lerman LO, Lerman A (2003). Endothelial dysfunction: a marker of atherosclerotic risk. Arterioscler Thromb Vasc Biol.

[b11-ehp0114-001636] Brook RD, Brook JR, Urch B (2002). Inhalation of fine particulate air pollution and ozone causes acute arterial vasoconstriction in healthy adults. Circulation.

[b12-ehp0114-001636] Brook RD, Brook JR, Rajagopalan S (2003). Air pollution: the “heart” of the problem. Curr Hypertens Rep.

[b13-ehp0114-001636] Brook RD, Franklin B, Cascio W, Hong Y, Howard G, Lipsett M (2004). Expert Panel on Population and Prevention Science of the American Heart Association. Air pollution and cardiovascular disease: a statement for healthcare professionals from the Expert Panel on Population and Prevention Science of the American Heart Association. Circulation.

[b14-ehp0114-001636] Burczynski ME, Lin HK, Penning JM (1999). Isoform-specific induction of a human aldo-keto reductase by polycyclic aromatic hydrocarbons (PAHs), electrophiles, and oxidative stress: implications for the alternative pathway for PAH activation catalyzed by human dihyrodiol dehydrogenase. Cancer Res.

[b15-ehp0114-001636] Campbell A, Oldham M, Becaria A, Bondy SC, Meacher D, Sioutas C (2005). Particulate matter in polluted air may increase biomarkers of inflammation in mouse brain. Neurotoxicology.

[b16-ehp0114-001636] Cao G, Alessio HM, Cutler RG (1993). Oxygen-radical absorbance capacity assay for antioxidants. Free Radic Biol Med.

[b17-ehp0114-001636] Cao G, Sofic E, Prior RL (1996). Antioxidant capacity of tea and common vegetables. J Agric Food Chem.

[b18-ehp0114-001636] Carluccio MA, Siculella L, Ancora MA, Massaro M, Scoditti E, Storelli C (2003). Olive oil and red wine antioxidant polyphenols inhibit endothelial activation. Antiatherogenic properties of Mediterranean diet phytochemicals. Arterioscler Thromb Vasc Biol.

[b19-ehp0114-001636] Chen L, Yang W, Jennison BL, Goodrich A, Omaye ST (2002). Air pollution and birth weight in northern Nevada, 1991–1999. Inhal Toxicol.

[b20-ehp0114-001636] Costa DL, Dreher KL (1997). Bioavailable transition metals in particulate matter mediate cardiopulmonary injury in healthy and compromised animal models. Environ Health Perspect.

[b21-ehp0114-001636] Crawford M (2000). Placental delivery of arachidonic and docosahexaenoic acids: implications for the lipid nutrition of preterm infants. Am J Clin Nutr.

[b22-ehp0114-001636] Cuevas AM, Germain AM (2004). Diet and endothelial function. Biol Res.

[b23-ehp0114-001636] Dejmek J, Selevan SG, Benes I, Solanksy I, Šrám RJ (1999). Fetal growth and maternal exposure to particulate matter during pregnancy. Environ Health Perspect.

[b24-ehp0114-001636] Dejmek J, Solansky I, Beneš I, Leníèek J, Šrám RJ (2000). The impact of polycyclic aromatic hydrocarbons and fine particles on pregnancy outcome. Environ Health Perspect.

[b25-ehp0114-001636] Delfino RJ, Sioutas C, Malik S (2005). Potential role of ultrafine particles in associations between airborne particle mass and cardiovascular health. Environ Health Perspect.

[b26-ehp0114-001636] Donaldson K, MacNee W (2001). Potential mechanisms of adverse pulmonary and cardiovascular effects of particulate air pollution (PM_10_). Int J Hyg Environ Health.

[b27-ehp0114-001636] Duvekot JJ, Cherlex EC, Pieters FAA (1995). Severely impaired growth is preceded by maternal hemodynamic maladaptation in very early pregnancy. Acta Obstet Gynecol Scand.

[b28-ehp0114-001636] Dvonch JT, Brook RD, Keeler GJ, Rajagopalan S, D’Alecy LG, Marsik FJ (2004). Effects of concentrated fine ambient particles on rat plasma levels of asymmetric dimethylarginine. Inhal Toxicol.

[b29-ehp0114-001636] Ehrenberg HM, Dierker L, Milluzzi C, Mercer BM (2003). Low maternal weight, failure to thrive in pregnancy, and adverse pregnancy outcomes. Am J Obsts Gynecol.

[b30-ehp0114-001636] Fall CHD, Yajnik CS, Rao S, Davies AA (2003). Micronutrients and fetal growth. J Nutr.

[b31-ehp0114-001636] Ford ES, Mokdad AH, Liu S (2005). Healthy Eating Index and C-reactive protein concentration: findings from the National Health and Nutrition Examination Survey III, 1988–1994. Eur J Clin Nutr 2005.

[b32-ehp0114-001636] Foster-Powell K, Holt SH, Brand-Miller JC (2002). International table of glycemic index and glycemic load values. Am J Clin Nutr.

[b33-ehp0114-001636] Fung TT, McCullough ML, Newby PK, Manson JE, Meigs JB, Rifai N (2005). Diet quality scores and plasma concentrations of markers of inflammation and endothelial dysfunction. Am J Clin Nutr.

[b34-ehp0114-001636] Fung TT, Rimm EB, Spiegelman D, Rifai N, Tofler GH, Willett WC (2001). Association between dietary patterns and plasma biomarkers of obesity and cardiovascular disease risk. Am J Clin Nutr.

[b35-ehp0114-001636] Ghio AJ, Cohen MD (2005). Disruption of iron homeostasis as a mechanism of biologic effect by ambient air pollution particles. Inhal Toxicol.

[b36-ehp0114-001636] Giugliano D, Nappo F, Coppola L (2001). Pizza and vegetables don’t stick to the endothelium. Circulation.

[b37-ehp0114-001636] Goerre S, Staehli C, Shaw S, Luscher TF (1995). Effect of cigarette smoking and nicotine on plasma endothelin-1 levels. J Cardiol Pharmacol.

[b38-ehp0114-001636] Gwynn RC, Thurston GD (2001). The burden of air pollution: impacts among racial minorities. Environ Health Perspect.

[b39-ehp0114-001636] Harding JE, Johnston BM (1995). Nutrition and fetal growth. Reprod Fertil Dev.

[b40-ehp0114-001636] Hartwig A, Asmuss M, Ehleben I, Herzer U, Kostelac D, Pelzer A (2002). Interference by toxic metal ions with DNA repair processes and cell cycle control: molecular mechanisms. Environ Health Perspect.

[b41-ehp0114-001636] Hennig B, Reiterer G, Majkova Z, Oesterling E, Meerarani P, Toborek M (2005). Modification of environmental toxicity by nutrients: implications in atherosclerosis. Cardiovasc Toxicol.

[b42-ehp0114-001636] Henriksen T, Clausen T (2002). The fetal origins hypothesis: placental insufficiency and inheritance versus maternal malnutrition in well-nourished populations. Acta Obsts Gynecol Scand.

[b43-ehp0114-001636] Hobel C, Culhane J (2003). Role of psychosocial and nutritional stress on poor pregnancy outcome. J Nutr.

[b44-ehp0114-001636] Hoffmann K, Schulze MB, Schienkiewitz A, Nothlings U, Boeing H (2004). Application of a new statistical method to derive dietary patterns in nutritional epidemiology. Am J Epidemiol.

[b45-ehp0114-001636] Hu FB (2002). Dietary patterns analysis: a new direction in nutritional epidemiology. Curr Opin Lipidol.

[b46-ehp0114-001636] Hwang SJ, Ballantyne CM, Sharrett AR, Smith LC, Davis CE, Gotto AM (1997). Circulating adhesion molecules VCAM-1, ICAM-1, and E-selectin in carotid atherosclerosis and incident coronary heart disease cases: the Atherosclerosis Risk In Communities (ARIC) study. Circulation.

[b47-ehp0114-001636] Ibald-Mulli A, Steiber J, Wichmann HE, Koenig W, Peters A (2001). Effects of air pollution on blood pressure: a population-based approach. Am J Public Health.

[b48-ehp0114-001636] Ibald-Mulli A, Timonen KL, Peters A, Heinrich J, Wolke G, Lanki T (2004). Effects of particulate air pollution on blood pressure and heart rate in subjects with cardiovascular disease: a multicenter approach. Environ Health Perspect.

[b49-ehp0114-001636] Institute of Medicine 1999. Toward Environmental Justice: Research, Education and Health Policy Needs. Washington DC:National Academy Press. 23035313

[b50-ehp0114-001636] Jansson JH, Nilsson TK, Johnson O (1991). von Willebrand factor in plasma: a novel risk factor for recurrent myocardial infarction and death. Br Heart J.

[b51-ehp0114-001636] Jarvi AE, Karlstrom BE, Granfeldt Y, Bjorck IE, Asp NG, Vessby BO (1999). Improved glycemic control and lipid profile and normalized fibrinolytic activity on a low-glycemic index diet in type 2 diabetic patients. Diabetes Care.

[b52-ehp0114-001636] Kant AK (1996). Indexes of overall diet quality: a review. J Am Diet Assoc.

[b53-ehp0114-001636] Keen CL, Clegg MS, Hanna LA, Lanoue L, Rogers JM, Daston GP (2003). The plausibility of micronutrient deficiencies being a significant contributing factor to the occurrence of pregnancy complications. J Nutr.

[b54-ehp0114-001636] Kennedy ET, Ohls J, Carlson S, Fleming K (1995). The healthy eating index: design and implications. J Am Diet Assoc.

[b55-ehp0114-001636] Kerver JM, Yang EJ, Bianchi L, Song WO (2003). Dietary patterns associated with risk factors for cardiovascular disease in healthy US adults. Am J Clin Nutr.

[b56-ehp0114-001636] Knottnerus JA, Delgado LR, Knipschild PG, Essed GG, Smits F (1990). Haematologic parameters and pregnancy outcome. A prospective cohort study in the third trimester. J Clin Epidemiol.

[b57-ehp0114-001636] Kristal AR, Vizenor NC, Patterson RE, Neuhouser ML, Shattuck AL, McLerran D (2000). Precision and bias of food frequency-based measures of fruit and vegetable intakes. Cancer Epidemiol Biomarkers Prev.

[b58-ehp0114-001636] Liao D, Heiss G, Chinchilli VM, Duan Y, Folsom AR, Lin HM (2005). Association of criteria pollutants with plasma hemostatic/inflammatory markers: a population-based study. J Expo Anal Environ Epidemiol.

[b59-ehp0114-001636] Linn WS, Gong H, Clark KW, Anderson KR (1999). Day-to-day particulate exposures and health changes in Los Angeles area residents with severe lung disease. J Air Waste Manag Assoc.

[b60-ehp0114-001636] Lopez-Garcia EMB, Fung TT, Meigs JB, Rifai N, Manson JE (2004). Major dietary patterns are related to plasma concentrations of markers of inflammation and endothelial dysfunction. Am J Clin Nutr.

[b61-ehp0114-001636] Mardones-Santander DF, Rosso DP, Uiterwaal D, Marshall DG (1999). Nutritional interventions to prevent intrauterine growth retardation: evidence from randomized controlled trials. Eur J Clin Nutr.

[b62-ehp0114-001636] Martin JA, Hamilton BE, Ventura SJ, Menacker F, Park MM (2002). Births: final data for 2000. Natl Vital Stat Rep.

[b63-ehp0114-001636] Martinez-Gonzalez MA, Sanche-Villegas A (2004). The emerging role of Mediterranean diets in cardiovascular epidemiology: monounsaturated fats, olive oil, red wine or the whole pattern?. Eur J Epidemiol.

[b64-ehp0114-001636] Mayne ST (2003). Antioxidant nutrients and chronic disease: use of biomarkers of exposure and oxidative stress status in epidemiologic research. J Nutr.

[b65-ehp0114-001636] McCully KS (1993). Chemical pathology of homocysteine. I. Atherogenesis. Am J Clin Lab Sci.

[b66-ehp0114-001636] Miller GJ (2005). Dietary fatty acids and the haemostatic system. Atherosclerosis.

[b67-ehp0114-001636] Misra DP (1996). The effect of pregnancy-induced hypertension on fetal growth: A review of the literature. Paediatr Perinat Epidemiol.

[b68-ehp0114-001636] Mohorovic L (2004). First two months of pregnancy—critical time for preterm delivery and low birthweight caused by adverse effects of coal combustion toxics. Early Hum Dev.

[b69-ehp0114-001636] Morrow JD, Hill KE, Burk RF, Nammour TM, Badr KF, Roberts LJ (1990). A series of prostaglandin F2-like compounds are produced *in vivo* in humans by a non-cyclooxygenase, free radical-catalyzed mechanism. Proc Natl Acad Sci USA.

[b70-ehp0114-001636] Nel AE, Diaz-Sanchez D, Ng D, Hiura T, Saxon A (1998). Enhancement of allergic inflammation by the interaction between diesel exhaust particles and the immune system. J Allergy Clin Immunol.

[b71-ehp0114-001636] O’Byrne DJ, DeVaraj S, Grundy SM, Jialal I (2002). Comparison of the antioxidant effects of concord grape juice flavonoids alpha-tocopherol on markers of oxidative stress in healthy adults. Am J Clin Nutr.

[b72-ehp0114-001636] Ordovas JM (2004). The quest for cardiovascular health in the genomic era: nutrigenetics and plasma lipoproteins. Proc Nutr Soc.

[b73-ehp0114-001636] Ostro B, Broadwin R, Green S, Feng WY, Lipsett M (2006). Fine particulate air pollution and mortality in nine California counties: results from CALFINE. Environ Health Perspect.

[b74-ehp0114-001636] Otsuka R, Watanabe H, Hirata K, Tokai K, Muro T, Yoshiyama M (2001). Acute effects of passive smoking on the coronary circulation in healthy young adults. JAMA.

[b75-ehp0114-001636] Panagiotakos DB, Pitsavos C, Chrysohoou C, Skoumas J, Masoura C, Toutouzas P (2004). Effect of exposure to secondhand smoke on markers of inflammation: the ATTICA study. Am J Med.

[b76-ehp0114-001636] Pekkanen J, Brunner EJ, Anderson HR, Tiittanen P, Atkinson RW (2000). Daily concentrations of air pollution and plasma fibrinogen in London. Occup Environ Med.

[b77-ehp0114-001636] Pearson TA, Mensah GA, Alexander RW, Anderson JL, Cannon RO, Criqui M (2003). Markers of inflammation and cardiovascular disease: application to clinical and public health practice: a statement for healthcare professionals from the Centers for Disease Control and Prevention and the American Heart Association. Circulation.

[b78-ehp0114-001636] Perera FP, Jedrychowski W, Rauh V, Whyatt RM (1999). Molecular epidemiologic research on the effects of environmental pollutants on the fetus. Environ Health Perspect.

[b79-ehp0114-001636] Perera FP, Whyatt RM, Jedrychowski W, Rauh V, Manchester D, Santella RM (1998). Recent developments in molecular epidemiology: a study of the effects of environmental polycyclic aromatic hydrocarbons on birth outcomes in Poland. Am J Epidemiol.

[b80-ehp0114-001636] Peters A, Doring A, Wichmann HE, Koenig W (1997). Increased plasma viscosity during an air pollution episode: a link to mortality?. Lancet.

[b81-ehp0114-001636] Peters A, Frohlich M, Doring A, Immervoll T, Wichmann HE, Hutchinson WL (2001). Particulate air pollution is associated with an acute phase response in men; results from the MONICA–Augsburg Study. Eur Heart J.

[b82-ehp0114-001636] Peterson J, Dwyer J (1998). Taxonomic classification helps identify flavonoid-containing foods on a semiquantitative food frequency questionnaire. J Am Diet Assoc.

[b83-ehp0114-001636] Pope CA, Burnett RT, Thurston G, Thun MJ, Callee EE, Krewski D (2004a). Cardiovascular mortality and long-term exposure to particulate air pollution. Circulation.

[b84-ehp0114-001636] Pope CA, Hansen ML, Long RW (2004b). Ambient particulate air pollution, heart rate variability, and blood markers of inflammation in a panel of elderly subjects. Environ Health Perspect.

[b85-ehp0114-001636] Porrini M, Riso P, Oriani G (2002). Spinach and tomato consumption increases lymphocyte DNA resistance to oxidative stress but this is not related to cell carotenoid concentrations. Eur J Nutr.

[b86-ehp0114-001636] Powers RW, Evans RW, Majors AK, Ojimba JI, Ness RB, Crombleholme WR (1998). Plasma homocysteine concentration is increased in preeclampsia and is associated with evidence of endothelial activation. Am J Obstet Gynecol.

[b87-ehp0114-001636] Prescott GJ, Lee RJ, Cohen GR, Elton RA, Lee AJ, Fowkes FG (2000). Investigation of factors which might indicate susceptibility to particulate air pollution. Occup Environ Med.

[b88-ehp0114-001636] Riediker M, Cascio WE, Griggs TR, Herbst MC, Bromberg PA, Neas L (2004). Particulate matter exposure in cars is associated with cardiovascular effects in healthy young men. Am J Respir Crit Care Med.

[b89-ehp0114-001636] Risom L, Moller P, Loft S (2005). Oxidative stress-induced DNA damage by particulate air pollution. Mutat Res.

[b90-ehp0114-001636] Ritz B, Yu F (1999). The effect of ambient carbon monoxide on low birth weight among children born in southern California between 1989 and 1993. Environ Health Perspect.

[b91-ehp0114-001636] Roberts JM, Balk JL, Bodnar LM, Belizan JM, Bergel E, Martinez A (2003). Nutrient involvement in preeclampsia. J Nutr.

[b92-ehp0114-001636] Rowley K, Walker KZ, Cohen J, Jenkins AJ, O’Neal D, Su Q (2003). Inflammation and vascular endothelial activation in an Aboriginal population: relationships to coronary disease risk factors and nutritional markers. Med J Aust.

[b93-ehp0114-001636] Salam MT, Millstein J, Li Y-F, Lurmann FW, Margolis HG, Gilliland FD (2005). Birth outcomes and prenatal exposure to ozone, carbon monoxide, and particulate matter: results from the Children’s Health Study. Environ Health Perspect.

[b94-ehp0114-001636] Saldiva PH, Clarke RW, Coull BA, Stearns RC, Lawrence J, Murthy GG (2002). Lung inflammation induced by concentrated ambient air particles is related to particle composition. Am J Respir Crit Care Med.

[b95-ehp0114-001636] Samet JM, Dominici F, Curriero FC, Coursac I, Zeger SL (2000). Fine particulate air pollution and mortality in 20 U.S. cities, 1987–1994. N Engl J Med.

[b96-ehp0114-001636] Schwartz J (2001). Air pollution and blood markers of cardiovascular risk. Environ Health Perspect.

[b97-ehp0114-001636] Seaton A, Soutar A, Crawford V, Elton R, McNerlan S, Cherrie J (1999). Particulate air pollution and the blood. Thorax.

[b98-ehp0114-001636] Smolkova B, Dusinka M, Raslova K, McNeill G, Spustova V, Blazicek P (2004). Seasonal changes in markers of oxidative damage to lipids and DNA: correlations with seasonal variation in diet. Mutat Res.

[b99-ehp0114-001636] Sorensen M, Dragsted LO, Hertel O, Knudsen LE, Loft S (2003). Personal PM_2.5_ exposure and markers of oxidative stress in blood. Environ. Health Perspect.

[b100-ehp0114-001636] Šrám RJ (1998). Effect of glutathione *S*-transferase M1 polymorphisms on biomarkers of exposure and effects. Environ Health Perspect.

[b101-ehp0114-001636] Šrám RJ (1999). Impact of air pollution on reproductive health. Environ Health Perspect.

[b102-ehp0114-001636] Šrám RJ, Binková B, Dejmek J, Bobak M (2005). Ambient air pollution and pregnancy outcomes: a review of the literature. Environ Health Perspect.

[b103-ehp0114-001636] Topinka J, Binkova B, Mrackova G, Stavkova Z, Benes I, Dejmek J (1997). DNA adducts in human placenta as related to air pollution and GSTMI genotype. Mutat Res.

[b104-ehp0114-001636] Trichopoulou A, Costacou T, Bamia C, Trichopoulos D (2003). Adherence to a Mediterranean diet and survival in a Greek population. N Engl J Med.

[b105-ehp0114-001636] Tseng M, DeVillis R (2000). Correlates of the “western” and “prudent” diet patterns in the US. Ann Epidemiol.

[b106-ehp0114-001636] United Nations International Children’s Fund (UNICEF) 2006. Progress for Children: Report Card for Nutrition. Available: http://www.unicef.org/progressforchildren/2006n4/index_lowbirthweight.html/ [accessed May 16, 2006].

[b107-ehp0114-001636] United States Department of Agriculture 1992 Composition of Foods: Raw, Processed, Prepared, 1963–1991. Washington, DC:U.S. Government Printing Office.

[b108-ehp0114-001636] Valkonen VP, Paiva H, Salonen JT, Lakka TA, Lehtimaki T, Laakso J (2001). Risk of acute coronary events and serum concentration of asymmetrical dimethylarginine. Lancet.

[b109-ehp0114-001636] Van Hoydonck PGA, Temme EHM, Schouten EG (2002). A dietary oxidative balance score of vitamin C, β-Carotene and iron intakes and mortality risk in male smoking Belgians. J Nutr.

[b110-ehp0114-001636] Virdis A, Schiffrin EL (2003). Vascular inflammation: a role in vascular disease in hypertension?. Curr Opin Nephrol Hypertens.

[b111-ehp0114-001636] Vollsett SE, Refsum H, Irgens LM, Emblem BM, Tverdal A, Gjessing HK (2000). Plasma total homocysteine, pregnancy complications and adverse pregnancy outcomes: the Hordland Homocysteine Study. Am J Clin Nutr.

[b112-ehp0114-001636] Wang X, Ding H, Ryan L, Xu X (1997). Association between air pollution and low birth weight: a community-based study. Environ Health Perspect.

[b113-ehp0114-001636] Willett W 1989. Nutritional Epidemiology. New York:Oxford University Press.

[b114-ehp0114-001636] Weikert C, Hoffmann K, Dierkes J, Zyriax BC, Klipstein-Grobusch KMB (2005). Homocysteine metabolism–related dietary pattern and the risk of coronary heart disease in two independent German study populations. J Nutr.

[b115-ehp0114-001636] Wilhelm M, Ritz B (2005). Local variations in CO and particulate air pollution and adverse birth outcomes in Los Angeles County, California, USA. Environ Health Perspect.

[b116-ehp0114-001636] Wright ME, Mayne ST, Stolzenberg-Solomon RZ, Li Z, Pietinen P, Taylor PR (2004). Development of a comprehensive dietary antioxidant index and application to lung cancer risk in a cohort of male smokers. Am J Epidemiol.

[b117-ehp0114-001636] Zanobetti A, Canner MJ, Stone PH, Schwartz J, Sher D, Eagan-Bengston E (2004). Ambient pollution and blood pressure in cardiac rehabilitation patients. Circulation.

